# Rapid Screening of Drug-Protein Binding Using High-Performance Affinity Chromatography with Columns Containing Immobilized Human Serum Albumin

**DOI:** 10.1155/2013/439039

**Published:** 2013-03-28

**Authors:** Ying-Fei Li, Xiao-Qiong Zhang, Wei-Yu Hu, Zheng Li, Ping-Xia Liu, Zhen-Qing Zhang

**Affiliations:** ^1^Beijing Institute of Pharmacology and Toxicology, Beijing 100850, China; ^2^Institute of Pharmacy, Medical School of Xiang Ya, Central South University, Changsha 410013, China; ^3^Hepatobiliary Surgery Department, The Affiliated Hospital of Medical College, Qingdao University, Qingdao 266003, China; ^4^Department of Pharmacy, Lushan Sanitarium of PLA, Jiujiang 332000, China

## Abstract

For drug candidates, a plasma protein binding (PPB) more than 90% is more meaningful and deserves further investigation in development. In the study, a high-performance liquid chromatography method employing column containing immobilized human serum albumin (HSA) to screen in vitro PPB of leading compounds was established and successfully applied to tested compounds. Good correlation (a coefficient correlation of 0.96) was attained between the reciprocal values (*X*) of experimentally obtained retention time of reference compounds eluted through HSA column and the reported PPB values (*Y*) with a correlation equation of * Y * = 92.03 − 97.01*X*. The method was successfully applied to six test compounds, and the result was confirmed by the conventional ultrafiltration technique, and both yielded equal results. However, due to the particular protein immobilized to column, the method cannot be applied for all compounds and should be exploited judiciously based on the value of the logarithmic measure of the acid dissociation constant (pKa) as per the requirement. If *α*1-acid glycoprotein and other plasma proteins could be immobilized like HSA with their actual ratio in plasma to column simultaneously, the result attained using immobilized column may be more accurate, and the method could be applied to more compounds without pKa limitation.

## 1. Introduction

When entering into plasma, most compounds bind rapidly to blood constituents. While the phenomenon of plasma protein binding (PPB) of a chemical is considered, it usually means the protein binding of drug molecules to blood components, such as albumin and  *α*1-acid glycoprotein [1]. The concentration of a free drug is primarily responsible for its pharmacological activity, safety, and tissue distribution. The extent of protein binding in plasma is, therefore, considered one of the important physiological factors affecting pharmacokinetic characteristics, such as clearance, volume of distribution, half-life, drug-drug interaction, and the pharmacological efficacy of a drug [2–6].

Conventionally, during the lead characterization stage, protein binding is investigated in rat, dog, monkey, and human plasma. these in vitro studies will help to characterize the therapeutic index for the selection of dose range in clinical trials. However, over the past decade, with the rapid rise in new molecular entities (NMEs) arising from computational lead discovery or modification of natural products, combinatorial chemistry, and high-throughput biological screening, an urgent need has arisen for the determination of the absorption, disposition, metabolism, and excretion properties of these NMEs or even “hit” at earlier stages in the drug discovery pipeline to speed up the selection of “ideal” drug candidates for further development. Back integration of key studies into the discovery phase enables earlier identification of potential drug metabolites/pharmacokinetics and safety liabilities [7]. This information increases the effectiveness of discovery scientists in lead selection, optimization, and enhancement of discovery biology and in many instances has been incorporated into criteria for compound advancement into the development phase [8, 9]. As one of important pharmacokinetic parameters, PPB of lead compounds should be screened at earlier stage. 

There are several in vitro methods for measuring the unbound drug concentration in plasma, including equilibrium dialysis, ultrafiltration, gel filtration, and albumin column [10]. Among them, equilibrium dialysis and ultrafiltration are the two most commonly used for determining the unbound drug concentrations in plasma or serum. However, given the need for earlier and more rapid evaluation of a larger number of lead compounds, it is necessary to establish a faster, higher-throughput, and less compound amount consuming approach to screen compound protein binding.

Compared with the conventional methods, chromatography-based methods employing columns immobilized with plasma proteins have been more and more popular over the years for the simplicity, specificity, and speed. Although plasma contains >60 different soluble proteins, among these, the major proteins that bind drug are albumin, the richest protein in plasma, and  *α*1-acid glycoprotein. The work in the area of immobilized HSA chromatography was initiated from Noctor and Wainer [11]. Henceforth, various strategies used to investigate the drug-protein interactions for the development of columns immobilized with HSA, including frontal analysis (frontal affinity chromatography), zonal elution, equilibrium dialysis combined with high-performance liquid chromatography (HPLC), and micellar liquid chromatography, have been reported [12–19]. However, all methods could not estimate the PPB of compounds accurately due to compounds binding to other proteins of plasma, in this way the PPB of compounds was often underestimated. Hence, that underestimation, the PPB of compounds was usually attained.

Being a quick means of in vitro PPB estimation based on human serum albumin-HPLC (HSA-HPLC), the method especially suits rapid screening of PPB for numerous leading compounds in early drug discovery phase. The present investigation reports an HSA-based HPLC method for drug-protein binding rapid screening of UV active compounds. An application of the method to six test compounds, was made and the results confirmed by ultrafiltration have also been applied.

## 2. Experimental

### 2.1. Chemicals and Reagents

**Figure 1:**
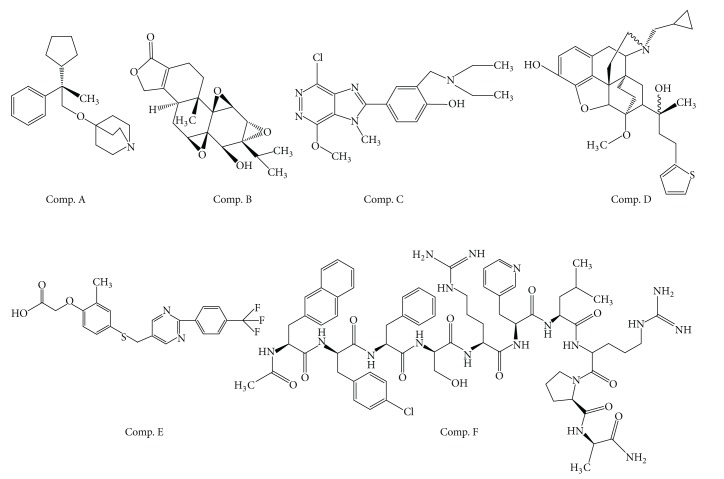
Chemical structures of six test compounds.

A set of commercially available reference compounds with a wide range of pKa was selected for this study. Caffeic acid, caffeine, catechuic acid, chlorphenamine maleate, chuanxiongzine hydrochloride, clomifene citrate, diazepam, diphenhydramine hydrochlo-ride, ferulic acid, fluconazole, hydrocortisone acetate, nefopam hydrochloride, ranitidine hydrochloride, phenacetin, phenytoin sodium, praziquantel, propranolol hydrochloride, progesterone, reserpine, rutoside, salicylic acid, sulfadiazine, quercetin, sulfadimidine, sulfamethoxazole, testosterone propionate, tolbutamide, vanillic acid, and warfarin sodium with purity of more than 98.6% (except rutin (92.5%) and warfarin sodium (92.6%)) were all supplied by the National Institutes for Food and Drug Control (Beijing, China). Compounds A, B, C, D, E, and F (Figure 1), with purity of more than 99%, were test compounds which experienced nonclinical pharmacokinetics evaluation as a new drug in our lab. A, B, and D were semisynthetic natural compounds.

Sodium chloride of analytical grade was purchased from Beijing Chemical Works (Beijing, China). HPLC grade methanol (USA) and acetonitrile (Trinidad) were purchased from Fisher Scientific Inc., and the other chemicals used were all of analytical reagent grade. HPLC grade water was prepared with a Direct-Q 3 UV water purifying system (Millipore, Bedford, MA, USA).

All solutions were kept in the refrigerator at 4°C. The solutions were filtrated through 0.22 *μ*m membrane (Millipore) before injection.

### 2.2. Instruments

The assay was performed on an Agilent 1100 series HPLC system (Agilent Technologies, Waldbronn, Germany) consisting of a quaternary pump, an autosampler, a vacuum degasser, and a UV absorbance detector set at 210 nm. 

### 2.3. Chromatographic Methods for Drug-Protein Binding Screening Using Immobilized HSA Columns

Compounds were dissolved with water or water-ethanol (with different ratio) to a concentration of 100 *µ*g/mL. The chromatographic peak was attained on a 5 *μ*m Chiral-HSA, 50 × 3 mm column (Chrom Tech, France), which was preceded by an on-line filter. The ChemStation for LC 3D software was used for data acquiring and handling. As a generic rapid method, an isocratic mobile phase was selected. To simulate physiology environment, a mobile phase consisting of 0.9% NaCl (pH 7.0) at a flow rate of 0.5 mL/min was used. 10 *µ*L aliquot of analyte solution was injected to HPLC to be analyzed.

### 2.4. PPB Experiment for Test Compounds by Ultrafiltration

The stock solution (1.0 or 2 mg/mL) of test compounds was prepared by dissolving appropriate amount of compounds in water-methanol (50 : 50, v/v) mixture. Working solution (1, 10 or 100 *μ*g/mL) was attained by diluted stock solution with water. A different volume of the working solution was added to a 2 mL eppendorf vial and evaporated to dryness under a stream of nitrogen in the thermostatically controlled water bath maintained at 55°C for about 20 min. Drug-free plasma (0.5 mL) was then added to it and vortexed for 5 min to get the final concentration of 10, 40, 200, 100, 2000, and 100 ng/mL for compounds A, B, C, D, E, and F, respectively. The mixture was incubated at 37°C for 30 min. Thereafter, 400 *µ*L aliquot of the sample was loaded into the preheated sample reservoir of Microcon YM-30 filter device (filter pore size 30 kDa) and centrifuged for 45 min at 5000 g and 37°C. The filtrate was analyzed for the drug content by HPLC-MS.

To estimate the nonspecific binding of the test drugs to the filter membrane, 500 *µ*L of the solution of each compound tested was centrifuged for 45 min at 5000 g in a Microcon YM-30 filter device. The concentration of the filtrate was analyzed by HPLC-MS. The nonspecific binding was <5% for each compound. The PPB by ultrafiltration was performed in triplicates for all the compounds studied.

### 2.5. pKa Calculation and Correlation Analysis

The pKa for all compounds except progesterone was calculated using ACD/labs 6.0. The pKa of progesterone was calculated by Pallas. Origin 6.0 was used to perform the plot and statistical analysis of the linear regressions. 

## 3. Results and Discussion

**Figure 2:**
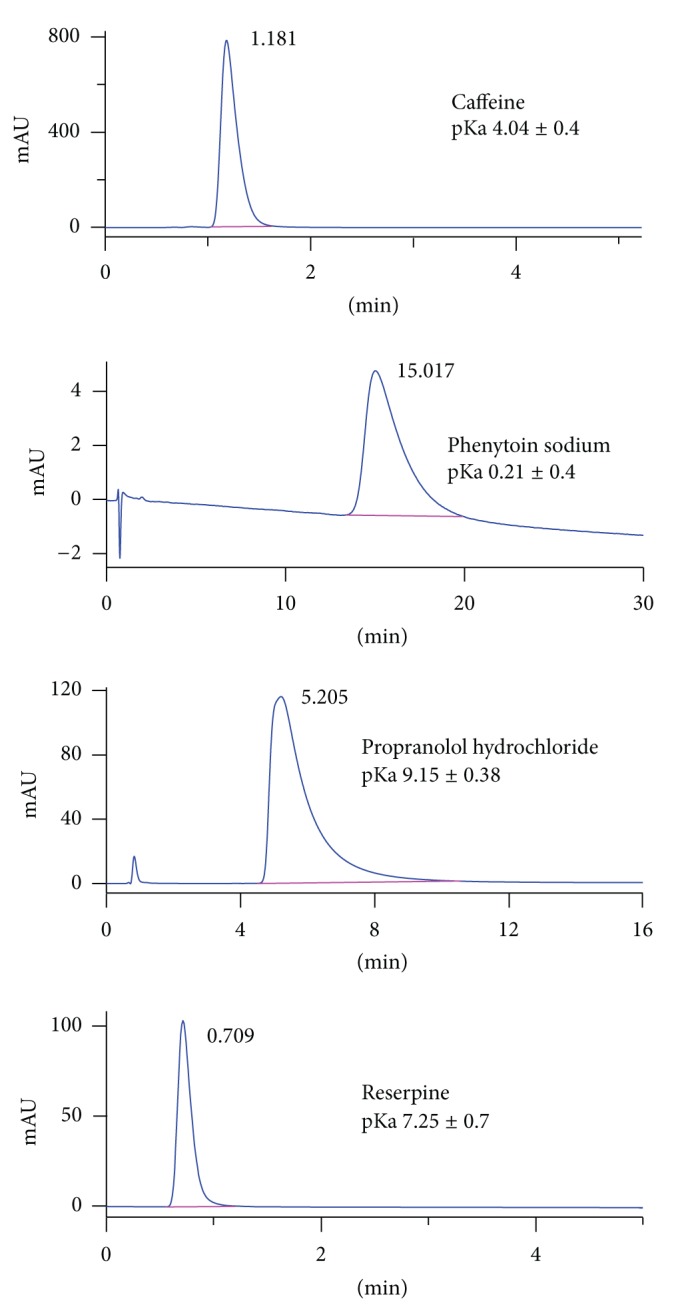
Representative chromatograms of some reference compounds analysed on HSA column.

Around 30 different compounds with PPB ranging from 11.5 to 98.0% were analyzed using immobilized HSA-HPLC. Most compounds with low PPB eluted at a lower retention time and exhibited sharp peak shapes as compared with the high protein bound compounds. Representative chromatograms are shown in Figure 2. There is one compound, clomifene citrate, that did not elute from HSA column to 240 min due to the strong binding. The related information about the reference compounds was listed in Table 1.

**Figure 3:**
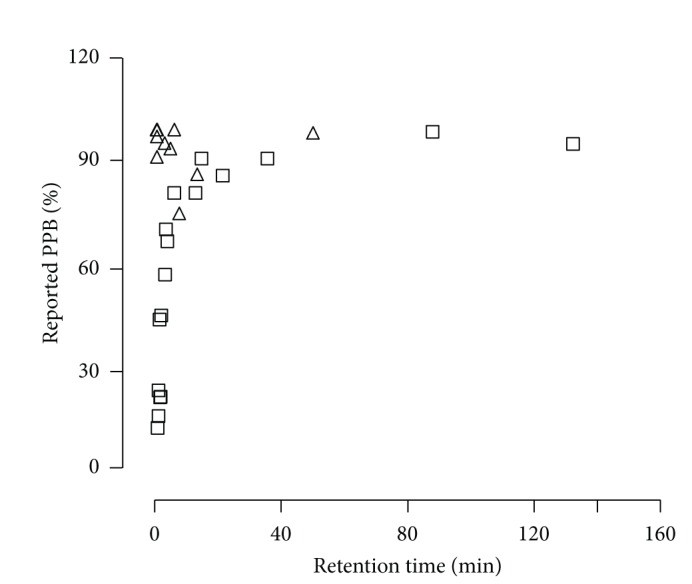
Scatter plot of retention time of reference compounds using immobilized HAS-HPLC and the corresponding reported PPB values. △: compounds with pKa < 7.0; □: compounds with pKa > 7.0.

**Figure 4:**
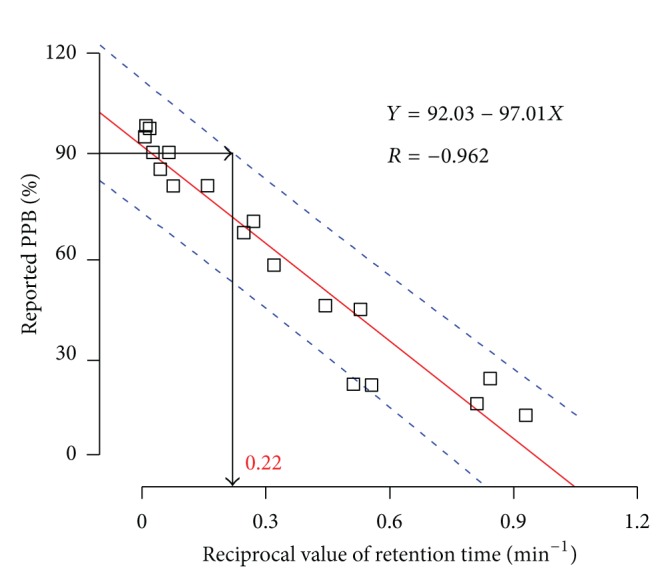
Correlation between reciprocal values of retention time of reference compounds (pKa < 7.0) by immobilized HAS-HPLC and the reported PPB values. The solid line was linear ship achieved by Origin 6.0 linear regression fit. The dash line represents 95% prediction limits.

Every compound binds to all the plasma proteins to a certain extent. Hence that for the data of all reference compounds, there is no manifest relationship between retention time (RT) and reported PPB of compounds as showed in Figure 3. Because albumin generally binds acidic drugs better while  *α*1-acid glycoprotein preferentially binds to basic drugs [1, 6], pKa less than 7.0 was used as a criterion for the drug binding to HSA mainly. However, for numerous leading compounds, it is difficult to know their actual pKa values. So the pKa values (Table 1) for drugs were predicted by ACD/Labs, one of the most accurate softwares to predict the pKa as reported [20]. Obviously, there is a hyperbola relationship for compounds with pKa less than 7.0 which mainly binds to HSA. A good linear correlation was attained between the reciprocal value of retention time and the reported PPB with a correlation equation of *Y* = 92.03–97.01*X* and coefficient correlation of 0.96 using linear regression fit of Origin 6.0 (Figure 4).

Some understanding of the possible binding characteristics of candidate molecules could supply valuable information in the strategies of the design process, so the linear relationship established above was used to rapidly predict the protein binding. An interaction is likely, and a clinical study should be performed to quantify the effects if the drug of interest has a PPB above 90%, and narrow therapeutic index, high hepatic extraction ratio, and especially intravenous administration will increase the possibility [21]. For leading compounds, PPB of 90% is an appropriate index to decide whether the PPB of a drug needs to be investigated using the method of equilibrium dialysis or ultrafiltration in the following development phase.

Based on the upper prediction limit with 95% confidence interval, the reciprocal value of retention time for the compound in the HSA column should be less than 0.22 when its protein binding was more than 90% (Figure 4). This means that the retention time of the drug was more than 4.55 min in the HSA column if its PPB was more than 90%. Although the pKa of diphenhydramine hydrochloride, rutoside, nefopam hydrochloride, progesterone, and propranolol hydrochloride is 8.76, 13.85, 9.16, 15.00, and 9.15, respectively, far more than 7.0, the retention time of the drugs in the HSA column was 6.23, 13.43, 7.87, 50.02, and 5.21 min, respectively, later than 4.50 min. The reported PPB of diphenhydramine hydrochloride, progesterone, and propranolol hydrochloride is 98%, 97%, and 90–95%, respectively. And the value for rutoside and nefopam was 80–90% and 71–76% close to 90%. Therefore, the compounds with retention time in the HSA column later than 4.55 min were potentially more than 90% regardless of the pKa value and need the further investigation.

The PPB attained by ultrafiltration was 78.2 ± 1.67%, 21.6 ± 2.30%, 51.0 ± 2.52%, 94.86 ± 0.56%, 99.5 ± 0.24%, and 97.2 ± 0.60%  for test compounds A, B, C, D, E, and F, respectively. The PPB of six compounds experienced nonclinical evaluation which was rapidly screened (Table 2) by the immobilized HSA-HPLC, and a confirmation with the result of the ultrafiltration was made. There were two compounds, D and E, with retention time more than 4.55 min which indicated that the PPB of these two compounds was more than 90%. The result of the ultrafiltration confirmed that the PPB was 95.0% and 99.5% for compounds D and E, respectively. For the left four compounds, only the pKa of compound C is less than 7.0 and the retention time of which is 1.75 min less than 4.55 min. So the PPB of compound C based on the HSA column was less than 90% and the PPB of compound C using ultrafiltration was 51.5%. The results were consistent with each other. However, the pKa of compounds A, F, and B was 9.49, 15.06, and 14.24, respectively, which is all more than 7.0. Due to binding to other constitutes of blood, the PPB of these drugs couldnot be predicted based on the retention time attained using the method of the HSA column.

Every compound binds to all the plasma proteins to a certain extent. The percentage protein binding obtained by the ultrafiltration method measures all the specific and nonspecific binding to all of the plasma components. Although the HSA-HPLC method measures both the specific and nonspecific binding to one particular protein and cannot reflect the binding to the other plasma proteins, it is still a quick means of in vitro PPB screening for lead compounds. If  *α*1-acid glycoprotein and other plasma proteins could be immobilized like HSA with their actual ratio in plasma to column simultaneously, the result attained using immobilized column may be more accurate, and the method could be applied to more compounds without pKa limitation. A further validation of the method is under way.

## 4. Conclusions

In accordance with the result of ultrafiltration, the HSA-HPLC method described in the present investigation could be applied in rapid screening lead compounds within 5 min in early drug discovery programs. It can be concluded that HSA-HPLC is suitable for the compounds that are designed to specifically or to some extent bind to HSA. The current commercial available HSA columns can be used in drug-protein binding screening while AGP columns are not as appropriate for such work. Further, using more analysis capacity HPLC-MS technique instead of HPLC, higher throughput for such studies will be acquired.

## Figures and Tables

**Table 1:** The retention time, reported PPB, and predicted pKa of reference compounds.

Compound	Retention time (min)	PPB from literature (%)	pKa based on ACD/labs
Caffeic acid	4.02	66.0	4.04 ± 0.40
Caffeine	1.18	22.5	0.63 ± 0.70
Catechuic acid	1.94	20.7	4.45 ± 0.10
Chlorphenamine maleate	5.93	37.5 (25–50)	3.77 ± 0.19
Chuanxiongzine hydrochloride	2.23	44.3	2.88 ± 0.50
Diazepam	132.21	94.5 (90–99)	3.40 ± 0.10
Ferulic acid	1.79	20.6	4.04 ± 0.40
Fluconazole	1.07	11.5 (11-12)	2.56 ± 0.12
Ranitidine hydrochloride	1.23	15.0 (12–18)	2.38 ± 0.70
Phenytoin sodium	15.02	90.0	0.21 ± 0.40
Praziquantel	12.97	80.0	−0.79 ± 0.20
Salicylic acid	21.81	85.0 (80–90)	3.01 ± 0.10
Sulfadiazine	1.88	43.0 (38–48)	1.57 ± 0.10
Sulfadimidine	6.22	80.0	1.55 ± 0.10
Sulfamethoxazole	3.68	69.5 (69-70)	1.39 ± 0.10
Tolbutamide	35.82	90.0	5.12 ± 0.50
Vanillic acid	3.11	56.3	4.45 ± 0.10
Warfarin	87.85	98.0	4.50 ± 1.00
Bendroflumethiazide	3.27	94.0	8.63 ± 0.40
Diphenhydramine hydrochloride	6.23	98.0	8.76 ± 0.28
Hydrocortisone acetate	0.73	90.0	13.46 ± 0.70
Rutoside	13.43	85.0 (80–90)	13.85 ± 0.70
Nefopam hydrochloride	7.87	73.5 (71–76)	9.16 ± 0.70
Phenacetin	3.19	30.0	14.57 ± 0.70
Progesterone	50.02	97.0	19.28*
Propranolol hydrochloride	5.21	92.5 (90–95)	9.15 ± 0.38
Quercetin	0.72	98.0	8.14 ± 0.60
Reserpine	0.71	96.0	7.25 ± 0.70
Testosterone propionate	0.74	98.0	9.63 ± 0.65

*Calculated by software of Pallas.

**Table 2:** Retention time, predicted pKa, experimental PPB, and result by HSA-HPLC of test compounds

Compound	Retention time (min)	pKa based on ACD/labs	PPB by ultrafiltration (%)	Screening result by HSA-HPLC (%)
Comp. A	0.76	9.49 ± 0.12	78.2	—
Comp. B	1.70	14.24 ± 0.60	21.6	—
Comp. C	1.75	1.17 ± 0.50	51.0	<90
Comp. D	4.87	9.46 ± 0.60	95.0	>90
Comp. E	>5.00	3.17 ± 0.10	99.5	>90
Comp. F	0.81	15.06 ± 0.46	97.2	—

—: compound is not appropriate for the method of HSA-HPLC.
